# The economic burden of amyotrophic lateral sclerosis for patients and families: a survey on out-of-pocket expenses and income loss in France

**DOI:** 10.1186/s13023-026-04326-1

**Published:** 2026-03-31

**Authors:** Claude Desnuelle, Philippe Couratier, Philippe Corcia, Thelma Arcelin, Camille Nevoret, Anne Duburcq, Sandrine Baffert, Sabine Turgeman

**Affiliations:** 1 ARSLA, Paris, France; 2 CRMR SLA & autres maladies du neurone moteur CHU de Limoges, Limoges, France; 3 CRMR SLA, CHU Tours, Tours, France; 4 CEMKA, 43 Bd Maréchal Joffre, Bourg la Reine, 92340 France

**Keywords:** ALS, Cost, Burden, Survey, France

## Abstract

**Introduction:**

Most published economic studies about the burden of amyotrophic lateral sclerosis (ALS) have measured direct and indirect costs but did not consider the perspective of patients and families.

**Materials and methods:**

The French ALS patient association, ARSLA, contacted 15 ALS reference centers to recruit a random sample of ALS patients stratified by age and severity accepting to participate in a structured interview survey. The objective was to document the out-of-pocket (OOP) expenses associated with ALS for patients and families in 2024.

**Results:**

Fifty patients with a mean age 61.5 years (SD 12) participated in the survey. The average time since diagnosis was 28.9 months (SD 26.8). Most patients had disabling motor impairment, half of them (*n* = 26, 52%) affected 4 limbs, 27 (54%) required noninvasive respiratory support and 11 (22%) nutritional support. A proportion of 48% of patients or caregivers reported Out-of-Pocket (OOP) expenses for mobility equipment, 50% for home modifications, 40% for vehicle adaptations, and 54% for paying for paid caregivers. The mean annual OOP expenses was €7,764 (SD 9,776) in total of which home adaptations averaged €3,074 (SD 6,583), personal vehicle adaptation €2,774 (SD 5,239) and paid caregivers €1,279 (SD 3,351). The factors statistically significant for higher overall OOP expenses were the duration of ALS since diagnostic (*p* = 0.026) and use of paid caregivers (*p* = 0.005). The support of family caregivers was almost systematic (88%), involving spouses (95.5%), children (22.7%) and relatives (13.6%). The mean annual loss of family income associated with professional change of patients and family caregivers was €7,633. When added to the OOP, the mean total annual economic burden was €15,397.

**Conclusion:**

Despite the full coverage of all ALS-related health expenditures within the French healthcare system and additional associated financial support, it remains a substantial economic burden for patients and families to manage this condition.

**Clinical trial registration:**

Not applicable.

## Introduction

Amyotrophic lateral sclerosis (ALS) is a neurodegenerative, progressive motor neuron disease leading to paralysis and death from respiratory failure typically within three to five years of diagnosis [1]. The disease-modifying treatments (DMTs) like riluzole used as first-line treatments do not reverse disease progression and increase survival only modestly [2, 3]. Consequently, clinical management focuses on treatment and care to alleviate symptoms, and to improve survival and health-related quality of life (HRQL). The intensity of care throughout the disease course leads to important healthcare resource use (HCRU) for patients with ALS, their caregivers, and healthcare systems [4]. This economic burden may be estimated by various types of cost of illness studies which are usually classified into two categories according to the way they are measuring costs. They may be restricted to direct medical and non-medical costs of HCRU or extend the scope to indirect costs from a societal perspective including productivity loss associated with premature mortality and work absenteeism in patients and caregivers. In most cases and even when the results are presented in terms of mean cost per capita per year, the perspective of patients’ household is rarely considered as such. For diseases which engage important support from family and external caregivers and often necessitate home and vehicle arrangements because of the presence of severe handicaps, this aspect presents a major interest. Thus, besides the need for formal medical care, most patients with ALS have a need for other healthcare and social services, such as rehabilitation, advanced healthcare services delivered at home, special transport services, and housing with special adaptations due to disability [5].

In a systematic literature review about the economic burden of ALS including 20 studies published in 2021 [6], 10 studies reported both direct as well as indirect costs. The total costs per person per year (PPPY) varied significantly between countries amounting from €9,741 in Greece to €114,605 in Australia. The non-medical costs accounted for the highest costs within two European studies of similar design: 60.0% in Greece [7] and 76.3% in Germany [8]. Most studies that covered jointly the direct and indirect costs of ALS have pointed out that direct medical cost constituted only a limited fraction of the whole cost. In Germany, on a total of €78 256, formal care represented 35.9%, direct non-medical costs (mainly constituted of informal care), 49.1% and productivity loss 15.0% [9]. In total, a very limited number of economic studies about ALS deliberately focused their analysis on the perspective of the patients and caregivers themselves and estimated the out-of-pocket part of the total costs incurred by the disease and its financial consequence as the level of the household. This observation was confirmed in another systematic review focusing on studies performed in the US [10]. The objectives of this study were to describe the consumption of resources not covered by public health insurance or other public fundings for handicap in France and estimate the corresponding annual cost to ALS patients and families. The total economic burden for patients comprises, on the one hand, all types of out-of-pocket expenses (OOP) incurred in the medical and non-medical management of patients, and, on the other hand, the loss of income due to patients’ and caregivers’ absence from work.

## Method

The initiative to document the economic burden for patients of ALS in France originated from the “Association pour la Recherche sur la SLA-Vaincre la maladie de Charcot” (ARSLA), a national association of ALS patients whose representatives took an active part in the design of the study [11].

### Patients’ selection and data collection

The collection of data was performed by a structured telephone survey on a stratified sample of ALS patients.

#### Regulatory permissions

In accordance with French medical data privacy laws, the protocol of the study, including the questionnaire, the information letter and the data collection process was submitted for approval to the “Comité d’Expertise pour les Recherches, les Etudes et les Evaluations dans le domaine de la Santé” (CESREES) (TPS 12301590) and the “Commission Nationale de l’Informatique et des Libertés” (CNIL) with an approval received on 01/05/2023 (ref 923144).

#### Patients’ selection and interview process

The selection of patients used a stepwise process as follows.

Patients with ALS are generally diagnosed and managed in France through a national network of 22 specialized units (ALS Reference and Referral centers) mostly located in regional university hospitals. A letter has been sent by ARSLA to each center asking them to recruit five patients presenting with a confirmed ALS diagnostic from their active patient list who might volunteer to participate in the survey. To ensure a diversity of patients’ profile and ALS severity, each center was asked to select a total of 5 patients stratified as follows: 1 walking patient, 1 wheelchair-bound patient, 1 ventilated patient and 2 patients under 65 of age. The objective was to recruit a total of 50 patients with sufficient regional diversity.

An information letter was then submitted to patients and caregivers who volunteered with all the details about the method used to collect data.

The telephone interview was performed by a single third party for each patient or caregiver who had agreed to participate within the regulatory conditions of confidentiality.

A detailed questionnaire was sent to patients by mail before the interview, to help them prepare their answers and collect the relevant documents. Interviews were conducted in 2024. The average duration of interviews was 40 min.

### Cost estimation

#### Coverage of direct medical costs

In France patients presenting with severe chronic disease belonging to a pre-defined list of 30 severe chronic diseases are eligible for full coverage of their disease-related healthcare expenditures [12]. This status (Long term Disease or LTD) is also used for “other disabling pathological states which include ALS requiring treatments lasting more than six months and which are particularly costly”. Under this status, a full reimbursement is provided for most items of outpatient and inpatient care but with some exceptions like extra fees for visits to specialists in private practice, patients’ contribution for acute care hospitalization for example. This status covers also in principle all transportation expenses for visits of patients to specialized centers as needed. It must be noticed that most French citizens benefit also from Complementary insurance (mutual or private insurance) that covers some remaining costs only partially covered by the public insurance.

The possibility to identify ALS patients in the French claims and hospital database (SNDS) through validated algorithms has been established [13]. Several studies are on-going to use this database to estimate the direct economic cost of ALS at a national level in the perspective of public health insurance on the totality of the prevalent population making the collection of direct medical costs from patients’ surveys of limited interest [14].

#### Other available financial support

In France, patients presenting with health-related impairment may benefit from various financial allowances that are summarized as follows for two of them [15, 16]. The AAH (“Allocation aux Adultes Handicapés” or Allowance for adults with disabilities) is a financial benefit granted to guarantee a minimum income for people of working age with disabilities. The benefit is means-tested. The examination of income includes partners’ income and depends on the number of dependent children. Another possible allowance is the “Allocation personalisée d’autonomie” or APA (Personal independence benefit). It is a financial social benefit paid out by the local authorities to older people aged at least 60 who require assistance to carry out everyday activities The benefit is also means-tested. On top of these allowances, income tax reductions may be presented to compensate for the cost of employment of a worker for home services, such as housekeeping, childcare, or tutoring, enabling taxpayers to claim a tax credit of 50% of the total cost with a cap of € 6,000 per tax household per year.

Due to the complexity of these allowances in terms of amount and period, it was judged non feasible to document in detail their amounts from patients that benefited from them. The instructions presented during interviews specified to document expenses not covered by these allowances or tax credit but neither receipt nor other factual justification were requested.

#### Data collected about non-medical costs

The items that were considered in the survey were covering the following categories: Home renovation, technical aids related to mobility (manual and electric wheelchair, walking aids, transfer disc etc.) and comfort (devices used at home, mattress, hospital beds, etc.), Personal vehicle-related expenses (change, modification of home access, etc.), professional care (paid caregivers), Communication devices, Partially or non-reimbursed ALS-related current expenditures (drugs, hygiene, etc.), Partially or non-reimbursed ALS-related visits to healthcare professionals (HCP). During the interviews with ALS patients, it was stipulated that OOP expenses of interest should not be directly or indirectly covered by any insurance either compulsory (public health insurance) or complementary or by any other kinds of public or private subsidies.

The utilization of non-reimbursed medical or non-medical resources was assessed retrospectively within different recall periods to reduce recall bias. For Home renovation, technical aids, personal vehicle-related expenses and purchase of Communication devices, the time since diagnostic at date of interview was considered. For professional care, patients were asked to document estimated monthly costs. Partially or non-reimbursed ALS-related current expenditures or HCP visits were assessed on an annual basis.

#### Loss of income

Loss of income of the household associated with professional activities cessation or reduction of the patient him/herself and the family caregivers was investigated based on interviewees declaration. They were asked to estimate it on a mean monthly basis. These values were then extrapolated to an annual amount, assuming a stable status over the period and added to annualized OOP estimates to estimate a total financial burden for patients/families. Specific questions related to household revenue per month at the time of interview were also asked as it may be a potential cost-driving factor on the levels of out-of-pocket expenses.

Costs were expressed in Euros (€) for the year 2024 (survey period).

### Statistical analysis

SAS^®^ V9.4 software program (North Carolina, USA) was used for statistical analysis. Continuous variables were described by mean, standard deviation (SD), median, minimum, and maximum, and categorical variables by numbers and percentages. For quantitative variables, a student t-test or analysis of variance was used when variables were normally distributed (non-significant Shapiro-Wilk test), otherwise, non-parametric tests such as Mann-Whitney *U-* test and Kruskall-Wallis test, as appropriate were applied to compare means between various strata. The p-value of < 0.05 was determined to be significant.

## Results

**Fig. 1:**
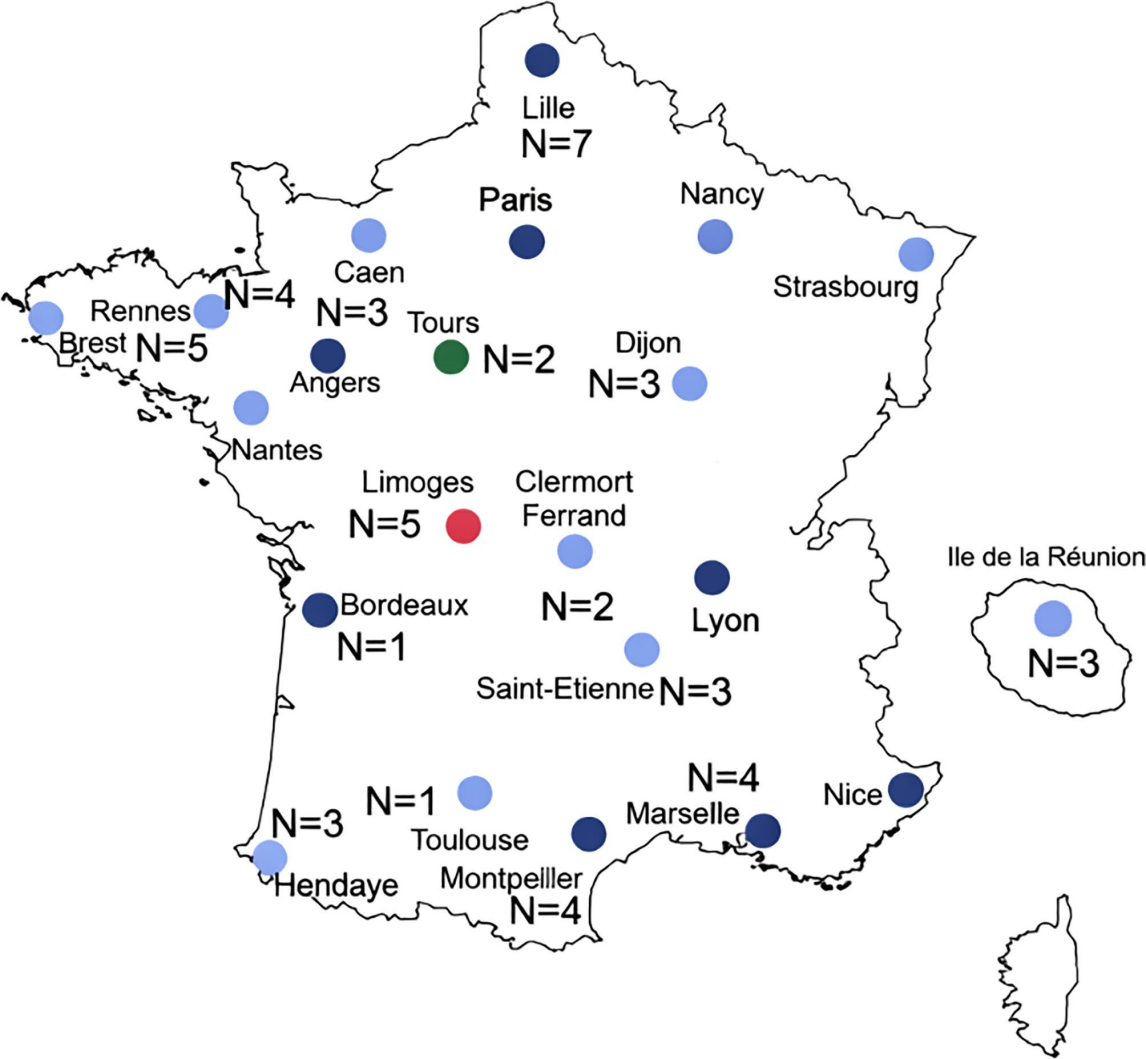
Location of patients having participated in the survey and SLA specialized units

The patient’s population was constituted of 50 patients recruited at 15 reference ALS centers located in various regions of metropolitan France (*n* = 47 patients) and overseas territories (*n* = 3 patients) (Fig. 1). A percentage of 54% of interviews were conducted with a family caregiver, in presence or not of the patient.

### Description of the population

The mean age of patients at survey completion was 61.5 years (median: 65.0, min 31- max 79) and 32 patients (64%) were males (Table 1). Mean disease duration since symptoms onset was 44.7 months (median 36.0) and since diagnostic 28.9 months (median 22.0). The mean delay between symptoms onset and diagnostic was then 15.8 months. The region of disease onset was limbs in 36 (72%) patients. 27 (54%) patients required respiratory support and 11 (22%) nutritional support. Most patients (*n* = 48, 92%) were living at their own home (vs. 2% living at a caregiver home).

**Table 1:** Socio-demographic and clinical characteristics of patients (*N* = 50)

Variable	
**Gender**	
Male/Female	32 (64%) / 18 (36%)
**Age at index date (years)**	
Mean (SD)	**61.5 +/- 12.0**
Median / min -max	65.0 / 31–79
**Disease duration since diagnostic (month)**	
Mean (SD)	28.9 (26.8)
Median / min -max	22.0 / 2–128
**Disease duration since symptoms onset (month)**	
Mean (SD)	44.7 (29.6)
Median / min -max	36.0 / 7.0 / 134
**Region of disease onset**	
Limb onset	36 (72%)
Other	14 (28%)
**Place of Residence**	
Personal home	46 (92.0%)
Caregiver home	4 (8.0%)
**Living with a partner/spouse**	38 (76.0%)
Alone	4 (8.0%)
Another situation	8 (16%)
**Nutritional supplementation**	11 (22.0%)
**Noninvasive ventilation**	27 (54.0%)
**Beneficiary from any financial support for handicap+**	18 (36.0%)

+ Allocation aux Adultes Handicapés (AAH), Allocation Personnalisée d’Autonomie (APA)

A total of 18 (36%) patients benefited from financial public support (AAH or APA) for their handicap.

### Patients and family employment and income loss

In Table 2, the employment status of patients and family caregivers and their evolution from date of ALS symptoms onset to date of interview is first presented. On the date of first symptoms onset, 24 (48%) patients were currently working (92% full-time) and 23 (46%) were retired.

**Table 2:** Employment status changes of patients and family caregivers and associated income loss (*N* = 50)

Employment status of patients	
**Employment status at symptoms onset**	
Employed	24 (48.0%)
Not employed	3 (6.0%)
Retired	23 (46.0%)
**Employment status at date of interview**	
Working	3 (6.0%)
Invalidity	13 (26.0%)
Retired or unemployed	26 (52.0%)
Sick leave	8 (16.0%)
**Involvement of patients’ relatives**	
**Are relatives helping you in daily life and for ALS management?**	
Any relative(s) (multiple response possible)	44 (88.0%)
* Spouse*	*42 (84.0%)*
* Parent(s)*	*6 (12.0%)*
* Children*	*10 (20.0%)*
* Other*	*4 (8.0%)*
**Professional activity of spouse before ALS diagnostic?**	
Spouse active	25 (59.5%)
* Full time*	*22 (88.0%)*
* Partial time*	*3 (12.0%)*
**Modification of professional activity of spouse?**	
Any modification	16 (32%)
* Stop completely*	*3 (18.7%)*
* On sick leave*	*4 (25%)*
* Go partial time*	*3 (18.7%)*
* Other types of modification*	*6 (37.5%)*
**Household income loss (€**)	
Loss of monthly household income associated with ALS	€ 636.1 (1,090)
mean (SD)	

At the time of interview, i.e. 44.7 months later (3.7 years), the situation of patients had dramatically evolved among those previously active. Only 3 (6%) of them were still working (including 1 full-time and 2 part-time), 13 (26%) in working age benefitted from a status of invalidity as defined by the French health insurance and 8 (16%) were in long term sick leave.

The involvement of patients’ relatives is described below. Most patients (*n* = 48, 88%) had one or several relatives as caregivers. It was mostly the spouse in 42 (95.5%), but also children (*n* = 10, 22.7%) or parents (*n* = 6, 13.6%). The employment of the spouse was deeply modified due to this involvement. Before ALS diagnosis, 25 (59.5%) of them were employed, full or part time. Among them 16 (64%) had to change their professional activity by stopping it completely (3), going part-time (3) and 4 of them were on sick leave.

The corresponding estimated annual loss of family (household) income was €7,633 on average in the whole population interviewed.

The average cumulative time spent per week by family caregivers was 115 h, of which the major part was related to spouse 105 h. For the sake of comparison with other published studies using a societal perspective, it was interesting to multiply the time of care provided by informal caregivers by the current statutory minimum wage in France (€10) and thus estimate the annual cost that would have risen if the care had been provided by professional caregivers, i.e. €59,840.

**Fig. 2:**
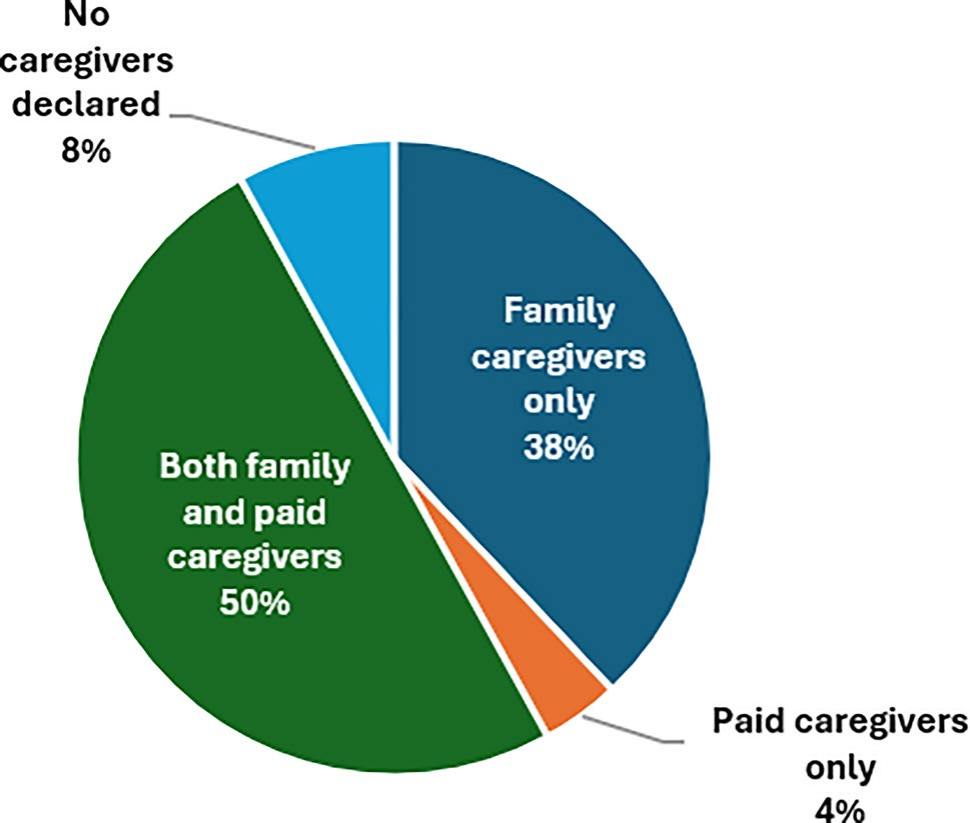
Family and paid caregivers’ involvement (*N* = 50)

Figure 2 provides a global view of the involvement of both family and paid caregivers. Only 4 (8%) patients declared themselves to have no caregivers. Most of the others had a mix of paid and family caregivers.

### Out-of-pocket expenses related to non-medical costs

In Table 3, a summary of out-of-pocket costs by category is presented. The percentages of patients having declared at least one item of expenses in each category are displayed and the corresponding mean annualized (SD) costs. The results are also presented as means (SD) for the total sample (50 patients) with or without expenses. Among them, 45 (90%) patients declared at least once an out-of-pocket expense and the mean annualized cost was €7,764 (SD 9,776).

**Table 3:** Annual non-medical direct cost incurred by ALS patients by category of expenditures (*N* = 50)

	Number of patients with at least one expenditure by category *N* (%)	Mean annualized cost - euros (SD)		Annualization method
		calculated on patients having declared at least one item	calculated on the whole sample	
**Home modification**	25 (50%)	€ 6,148 (8,295)	€ 3,074 (6,583)	*Time since diagnosis*
**Technical aids**				*Time since diagnosis*
Mobility (manual and electric wheelchair, walking aids, transfer disc etc.)	24 (48%)	€ 687 (869)	€ 330 (689)	
Comfort (devices used at home, mattress, etc.)	19 (38%)	€ 251 (551)	€ 95 (356)	
**Personal vehicle**				
(change, modification of home access, etc.)	20 (40%)	€ 6,935 (6,356)	€ 2,774 (5,239)	*Time since diagnosis*
**Informal care (paid caregivers)**	27 (54%)	€ 2,368 (4 300)	€ 1,279 (3,351)	*Monthly expenditures multiplied by 12*
**Communication devices**	6 (12%)	€ 465 (362)	€ 56 (192)	*Time since diagnosis*
**Partially or non-reimbursed ALS-related current expenditures** (drugs, hygiene, etc.)	15 (30%)	€ 372 (385)	€ 112 (268)	*Assumed to be annual as declared*
**Partially or non-reimbursed ALS-related visits to healthcare professionals**	33 (66%)	€ 66 (71)	€ 44 (66)	*Assumed to be annual as declared*
**Total**	**45 (90%)**	**€ 8**,**627 (9**,**940)**	**€ 7**,**764 (9**,**776)**	

#### Home modification costs

The most important contribution to these expenses was devoted to home modifications intended to facilitate the daily life of a person with handicap. Home renovations were reported by 50% of patients (*n* = 25) and the mean annual out-of-pocket costs per patient was €3,074 (SD 6,583). It included bathroom renovations in 24% of them (€6,956), stair-lift installation reported by 14% (€8,418), home automation system by 6% (€3,338), ramp installation by 8% (€212) and door enlargement (10%, €2,056). Five respondents (10%) reported having carried out home extension/renovation work, with an average cost of €48,472.

#### Personal vehicle change

The second item, mostly contributing to the total cost, was related to expenses devoted to change or adaptation of personal vehicle. It included, for example, the installation of a side lift or of a swivel car seat. This was declared by 20 (40%) respondents with an average annualized expense of €6,935 (SD 6,356) for them or €2,774 (SD 5,239) for the whole sample.

#### Paid caregivers

In 27 patients (54%) the use of one or several paid caregivers was reported, of which 20 (74%) as home help and in 8 (30%) as a nursing assistant.

#### Technical aids

The mean annual out-of-pocket costs per ALS patient for mobility devices like electric or manual wheelchair, walking aids, transfer disc was €330 (SD 689). Comfort devices like hospital beds and specific mattress purchases accounted for a mean €95 (SD 356) on the whole sample.

#### Purchased of partially or non-reimbursed products or services

Non-reimbursed prescription drugs like Okimus (treatment for nighttime muscle cramps), vitamins, etc. were purchased by 26% of patients. Cosmetic or dermatological products (moisturizers, ointments, massage oils, etc.) by 26% of them, hygiene products (diapers, tissues, etc.) by 42%, nutrition products (dietary supplements) by 4% and adapted clothing (elastic/loose-fitting clothing, pajamas, slippers, etc.) by 16%. The corresponding mean annual out-of-pocket costs per patient was €112 (SD 268).

Other types of expenses were related to ALS-related non-reimbursed transportation to visit expert centers or other healthcare professionals.

#### Partially or non-reimbursed ALS-related visits to healthcare professionals

Non reimbursed visits to HCP like chiropractor, pedologist, sophrologist, relaxation therapist, concerned 33 (66%) patients for a mean annual cost of €44 (SD 66).

### Factors affecting out-of-pocket expenses

Independent cost-driving factors for direct OOP expenses are presented in Table 4. Statistically significant higher costs were associated with disease duration since diagnostic (*p* = 0.00236) and the use of paid caregivers (*p* = 0.0005). The monthly household income before ALS diagnostic was also discriminant despite not reaching statistical significance (*p* = 0.0701). It is noticeable that the variables that document the severity of the disease like the number of limbs affected, the use of nutritional supplementation or of non-invasive ventilation were non discriminant. The same was found for the demographic characteristics of patients like age group and gender and for the benefit from additional financial support for handicap (AAH or APA).

**Table 4:** Annual out-of-pocket costs stratified by potential cost-driving factors (*N* = 50)

Potential cost-driving factors	Mean cost (annualized) Euros Mean (95% CI)	*p*-value ^b^
**Gender**		0.6785
Male (*n* = 32)	8,038 (4,488–11,588)	
Female (*n* = 18)	7,277 (3,051–11,502)	
**Age (years)**		0. 8571
< 55 (*n* = 12)	8,013 (2,671–13,355)	
55–69 (*n* = 23)	6,297 (3,412–9,182)	
> 69 (*n* = 15)	9,814 (3,061–16,567)	
**Nutritional supplementation**		0.9253
Yes (*n* = 11)	6,176 (2,038–10,314)	
No (*n* = 39)	8,212 (4,929–11,494)	
**Noninvasive ventilation**		0.6264
Yes (*n* = 27)	8,107 (4,322–11,893)	
No (*n* = 23)	7,361 (3,409–11,313)	
**Disease duration since diagnostic (month)**		0.0236 ++
< 22 (*n* = 24)	5,543 (1,700–9,385)	
≥ 22 (*n* = 26)	9,814 (6,101–13,527)	
**Disease duration since first symptoms (month)**		0.1147
< 36 (*n* = 23)	6,849 (2,239–11,459)	
≥ 36 (*n* = 27)	8,543 (5,363–11,723)	
**Number of limbs currently affected**		0.2322
4 (*n* = 26)	9,468 (5,131–13,805)	
< 4 (*n* = 24)	5,918 (2,860–8,975)	
**Beneficiary from any financial support for handicap** (AAH or APA)		0.6637
Yes (*n* = 18)	7,669 (4,611–10,726)	
No (*n* = 32)	7,933 (2,597–13,269)	
**Use of paid carers**		0.0005 ++
Yes *n* = 23	12,363 (7,479–17,246)	
No *n* = 27	4,150 (1,914–6,386)	
**Household monthly income before ALS diagnosis (€)** ^**a**^		0.0701 +
** <** 3 270 (*n* = 19)	4,244 (1,707–6,782)	
≥ 3 270 (*n* = 19)	11,789 (6,255–17,323)	

^a^ Missing data *N* = 12 ^b^ p -values estimated by Mann-Whitney U -test or Kruskal-Wallis test, where applicable *p* < 0.05 significant

++ highly significant +nearly significant

## Discussion

This is the first French study that estimated the economic burden of ALS to patients and relatives. The sample size of this study was limited but included a broad patient population recruited in multiple centers covering the entire country which should guarantee a sufficient level of representativity.

Our perspective included the so-called “direct non-medical costs” as well as the loss of revenue of patient’s household associated with patients’ disability and family caregivers’ modifications of professional activities. The direct medical costs were not investigated in this study as a nation-wide claims database (Systeme National des Données de Santé or SNDS) may be used in France to identify ALS patients and estimate the direct medical cost of their management with different studies on-going [13, 14, 17, 18].

Our results suggest that, even with the ‘generosity’ of the French health insurance system — considered one of the top six in the 38 OECD countries in terms of healthcare expense coverage — OOP expenses remained a significant financial burden for families [19]. The proportion of household spending on healthcare goods and services provides an overall assessment of the financial burden of out-of-pocket (OOP) expenditure. In 2021, it was 2.2% in France, compared to around 3% across OECD countries, but above 5% in Portugal, Switzerland, and South Korea. This situation explains why the level of out-of-pocket (OOP) expenses for partially or non-reimbursed ALS-related current expenditure and visits to healthcare professionals (HCPs) was relatively low. Our definition of OOP was broader than the OECD’s as it included non-medical costs that were outside the scope of this international comparison. Regarding disability allowances, international comparisons are much more difficult to synthetize due to their complexity which makes it very difficult to put our results into perspective [20].

### Comparisons with similar studies

The patient’s perspective used in this study was different from the usually recommended methodologies for cost-of-illness studies distinguishing only third-party payer and societal perspectives [6]. Consequently, a very limited number of economic studies on ALS has been performed in this specific perspective aside from a seminal Canadian study [21]. In this study performed in Ontario in 2012/2013, the mean annual OOP direct non-medical cost was estimated $(Canada)19,574 or €8,309 after conversion using Purchasing-power parity and inflation to 2020 [6], which was similar to our estimate of €7,764. The most important types of OOP cost were home renovation, mobility and professional care (paid caregivers) which were also in line with our results. The main cost-driving factors of the OOP costs were use of paid caregivers and limb predominance like in this study. The mean annual income lost by patient and family was estimated $(Canadian) 41,061 or €17,430 i.e. 2.3 times higher than our estimate of €7,633.

The comparison of our results with other ALS cost of illness studies raises several difficulties due to differences in measurement methods, time horizon and healthcare expenses coverage.

The calculations of *caregiving costs* present, for example, different methodological approaches. They may include not only the cost of paid caregivers as an OOP for the patients but also an indirect estimate of the time of care of family caregivers multiplied by the statutory minimum wage following a human capital approach and a societal perspective. An estimate of the annual direct non-medical cost for Germany in 2018 [9] was €38,412 of which €36,152 was for caregiving including both paid and family caregivers. This result was in the same order of magnitude as previously found in 2010 [8] with 36,380€ in the same country. By using a similar method on our data, we estimated this annual cost of informal care at a higher level with €59,840. By using such a human capital approach, these costs constitute a substantial part of the ALS cost but are difficult to interpret and may be controversial methodologically [22].

In a systematic review about ALS economic burden issued in 2020, the authors could extract all predefined direct cost components in seven studies [6]. In six of them the non-medical costs amounting to €16,301 in two studies performed in the US [23, 24], €27,629 in a study in Spain [25], and €24,484 in South Corea [4], which accounted for the biggest part of the total direct costs. But the heterogeneity of cost components makes interpretation difficult. in the studies conducted in Spain [25] and South Korea [4] the non-medical costs included costs for transportation, informal care as well as formal care in case of the South Korean study.

## Limitations

This study has several limitations. The small sample size of this study resulted in poor statistical power, leading to uncertainty in our estimates in terms of confidence intervals.

### Absence of clinical description of ALS staging

In many studies initiated by specialized clinical centers, patients were described in terms of severity by classical clinical tools like the revised Amyotrophic Lateral Sclerosis Functional Rating Scale (ALSFRS-R) which measures the level of impairment of distinct motor functions (bulbar, fine and gross motor and respiratory function [26]. Other tools were also currently used as the King’s clinical staging system for ALS [27], and the Milan Torino Staging system for ALS (MiToS) [28]. We did not collect this information for regulatory reasons and our evaluation of the severity of ALS was based only on declaration of patients on a limited number of questions.

### Selection and memory biases

The questions used for this study were detailed, including over 100 items. This might have led to a selection bias in favor of more motivated, educated, or less severely affected patients. Even though we tried to minimize recall bias by using different recall periods, it may have still occurred and led to cost underestimation. Another possible bias is about the consideration of extra financial support like AAH and APA which may have been underestimated in the questions related to household income loss.

## Conclusion

Despite the full coverage of ALS within the French healthcare system and associated financial support, it remains a substantial economic burden for patients and families.

## Data Availability

The datasets used and/or analysed during the current study are available from the corresponding author on reasonable request.
